# Icariin Prevents H_2_O_2_-Induced Apoptosis via the PI3K/Akt Pathway in Rat Nucleus Pulposus Intervertebral Disc Cells

**DOI:** 10.1155/2017/2694261

**Published:** 2017-04-27

**Authors:** Xiangyu Deng, Sheng Chen, Dong Zheng, Zengwu Shao, Hang Liang, Hongzhi Hu

**Affiliations:** ^1^Department of Orthopaedic Surgery, Union Hospital, Tongji Medical College, Huazhong University of Science and Technology, Wuhan 430022, China; ^2^Tongji Medical College, Huazhong University of Science and Technology, Wuhan 430022, China

## Abstract

Icariin is a prenylated flavonol glycoside derived from the Chinese herb* Epimedium sagittatum.* This study investigated the mechanism by which icariin prevents H_2_O_2_-induced apoptosis in rat nucleus pulposus (NP) cells. NP cells were isolated from the rat intervertebral disc and they were divided into five groups after 3 passages: (A) blank control; (B) 200 *μ*M H_2_O_2_; (C) 200 *μ*M H_2_O_2_ + 20 *μ*M icariin; (D) 20 *μ*M icariin + 200 *μ*M H_2_O_2_ + 25 *μ*M LY294002; (E) 200 *μ*M H_2_O_2_ + 25 *μ*M LY294002. LY294002 is a selective inhibitor of the phosphoinositide 3-kinase (PI3K)/Akt signaling pathway. NP cell viability, apoptosis rate, intracellular reactive oxygen species levels, and the expression of AKT, p-AKT, p53, Bcl-2, Bax, caspase-3 were estimated. The results show that, compared with the control group, H_2_O_2_ significantly increased NP cell apoptosis and the level of intracellular ROS. Icariin pretreatment significantly decreased H_2_O_2_-induced apoptosis and intracellular ROS and upregulated p-Akt and BCL-2 and downregulated caspase-3 and Bax. LY294002 abolished the protective effects of icariin. Our results show that icariin can attenuate H2O2-induced apoptosis in rat nucleus pulposus cells and PI3K/AKT pathway is at least partly included in this protection effect.

## 1. Introduction

Low back pain (LBP) is a frequent musculoskeletal disorder worldwide that affects approximately 70% of the adult population sometime in their lives, and most result in musculoskeletal disability. LBP can cause an enormous economic burden every year. The societal cost (converted into 2008 prices) of back pain is estimated at £12.3 billion in the United Kingdom (£1.6 billion for direct healthcare resources, £1.6 billion related to informal care, and indirect costs of £9.1 billion through loss of productivity due to morbidity) and €16.5–50 billion in Germany [1–7]. Therefore, LBP is a major disease that severely impacts human health and results in an enormous strain on limited medical resources [6].

Intervertebral disc (IVD) degeneration is considered to be one of the main reasons for LBP [8]. Nucleus pulposus (NP) cells are of critical importance in maintaining the biomechanical properties of IVDs. A decreased number of NP cells and changes in the extracellular matrix composition are early pathologic signs of IVD degeneration. Moreover, reversal of IVD degeneration is almost impossible due to the poor self-repairing ability of NP tissues.

Icariin is one the most frequently prescribed medicinal herbs in traditional Chinese medicine. Qianggu Capsule, which contains icariin, is used to treat postmenopausal and posttraumatic osteoporosis. An increasing number of articles have reported that icariin possesses multiple biological activities, such as improvement of drug resistance to chemotherapy [9], tumor-suppression, and antioxidation. Additionally, we find that icariin has an initiative pathophysiological effect on the reversal of IVD degeneration. We speculate that this results from a protective effect of the icariin on NP cells. Icariin can prevent apoptosis and oxidative stress in several cell models. Because the selective phosphoinositide 3-kinase (PI3K) inhibitor, LY294002, can reverse this effect; the protection seen with icariin appears to occur through activation of the PI3K/Akt signaling pathway [10], implying that icariin activates this pathway.

In the current study, H_2_O_2_ was used to induce apoptosis of NP cell with or without pretreatment with icariin. Our findings suggest that H_2_O_2_ induces apoptosis in NP cells and icariin prevents it through the PI3K/AKT signaling pathway. Our results provide an initiative method for the treatment of IVD degeneration disease.

## 2. Materials and Methods

### 2.1. General Supplies

Instruments, reagents, and experimental animals were provided by the animal center of Tongji Medical College and Huazhong University of Science and Technology. H_2_O_2_ was purchased from Thermo Fisher Scientific (Waltham, MA, USA). Icariin (purity ≥ 98%) was purchased from Nanjing Zelang Pharmaceutical Technology (Nanjing, China). Fetal bovine serum was purchased from Thermo Fisher. F12-Dulbecco's modified Eagle medium was purchased from HyClone (Logan, UT, USA). Cell counting kit-8 (CCK8) was purchased from Kaiji Bioengineering Institute (Jiangsu, China). LY294002 was purchased from Sigma-Aldrich (St. Louis, MO, USA). The reactive oxygen species (ROS) detection kit was purchased from Nanjing Jiancheng Bioengineering Institute (Nanjing, China). The Annexin V-FITC/propidium iodide detection kit was purchased from Nanjing KeyGen Biotech (Nanjing, China). *β*-Actin, Bcl-2, bax, caspase-3, phospho(p)-Akt, rabbit monoclonal antibodies, and the p53, Akt mouse monoclonal antibody, were purchased from Abcam (Cambridge, UK). Goat anti-rabbit and goat anti-mouse IgG were purchased from Proteintech (Wuhan, China). Microplate Reader was purchased from Thermo. Inverted fluorescence microscope was from Olympus, Japan.

### 2.2. Culture and Synchronization of the NP Cells and the Detection of Cell Density and Morphology

The density and morphology of NP cells under different treatments were observed and photographed with an inverted phase-contrast microscope. NP cells were isolated using about 200 g NP tissue of rats. Briefly, NP tissue was aseptically removed in a Petri dish containing 0.25% (w/v) type II collagenase and cut into pieces 0.1 mm × 0.1 mm. Then samples were digested with 0.25% (w/v) type II collagenase for 15–20 min and serum was used to stop the reaction. After centrifugation at 1200 rpm for 7 min, the supernatant was discarded and the pellet was resuspended in F12-Dulbecco's modified Eagle medium supplemented with 20% fetal bovine serum, 100 U/mL penicillin, and 100 mg/L streptomycin. Cell cultures were maintained at 37°C and 5% CO_2_. Medium was changed 3–5 days later when the cells had been attached and then changed every other day. When NP cells reached approximately 80% confluence, each primary culture was subcultured at a 1 : 3 ratio with a 0.25% (w/v) trypsin solution.

### 2.3. Experimental Protocols

First cells were tested for the ability of icariin to activate the PI3K/AKT pathway. Kinetics of the phosphorylation of AKT were estimated by Western blot analysis at 0 h, 1 h, 2 h, 3 h, 4 h, and 5 h. Other cells were randomly separated into five groups with at least three replicates: (A) blank control; (B) 200 *μ*M H_2_O_2_; (C) 20 *μ*M icariin + 200 *μ*M H_2_O_2_; (D) 20 *μ*M icariin + 25 *μ*M LY294002 + 200 *μ*M H_2_O_2_; (E) 25 *μ*M LY294002 + 200 *μ*M H_2_O_2_. Treatment with LY294002, icariin, and H_2_O_2_ was performed for 2 h, 24 h, and 6 h, respectively.

### 2.4. Detection of Icariin Cytotoxicity and Cell Viability and Proliferation

NP cells at passage 3 were replated in 96-well plates at a density 1 × 10^5^ cells per well, and the culture medium was plated after synchronization. Cells were then treated with icariin for 24 h at various concentrations (0.1, 0.5, 1, 5, 10, 20, 40, and 50 *μ*M). Cell viability was detected according to the instructions of the CCK8 assay. Then cells were treated according to the aforementioned experimental groupings. Cell viability was detected according to the manufacturer's instructions.

### 2.5. Apoptosis Assay

Cells were harvested and washed with PBS twice at 4°C. Next, cells were resuspended in 200 *μ*L of binding buffer and incubated with 10 *μ*L of Annexin V-FITC solution (15 min, room temperature) in the dark. Then cells were incubated with 10 *μ*L PI and 300 *μ*L Binding Buffer and immediately analyzed in a BD FACSCalibur cytometer to separate living cells, apoptotic cells, and necrotic cells in different periods.

### 2.6. Detection of Intracellular ROS Levels by Flow Cytometry

Cells were treated differently according to the aforementioned experiment grouping design. Then 200 *μ*L of culture medium from each group was gathered to detect intracellular ROS levels. Experimental steps were strictly executed according to the manufacturer's instructions.

### 2.7. Expression of Akt, p-Akt, p53, Bcl-2, Bax, and Caspase-3 by Western Blot Analysis

Proteins were extracted according to the instructions of the Total Extraction Sample Kit. Equal amounts of proteins (10 *μ*g) were loaded onto 10% sodium dodecyl sulfate polyacrylamide gels, electrophoresed, and then transferred to polyvinylidene fluoride membranes. The membranes were incubated with 5% nonfat milk for 2 h followed by incubation with primary antibodies overnight at 4°C (0.5 *μ*g/mL Akt, p53, p-Akt, Bcl-2, and caspase-3; 1 : 5000). After washing in TBST, membranes were incubated with the secondary antibody for 1.5 h at room temperature (rabbit anti-mouse or goat anti-rabbit, 1 : 5000). Bands were visualized by incubating with enhanced chemiluminescence reagent for 2 min after membranes were washed with TBST. Densitometry of p-Akt, Akt, p53, Bcl-2, and bax as well as caspase-3 levels was performed using ImageJ software (National Institutes of Health, Bethesda, MD, USA).

### 2.8. Statistical Analysis

Data are presented as means ± standard deviation. For group-wise comparisons, a one-way ANOVA with the LSD or Dunnett's* T*3 test was performed using SPSS 19.0 (IBM, Chicago, IL, USA). Values were considered significantly different for *p* < 0.05.

## 3. Results

### 3.1. H_2_O_2_ Induced Apoptosis in NP Cells

**Figure 1:**
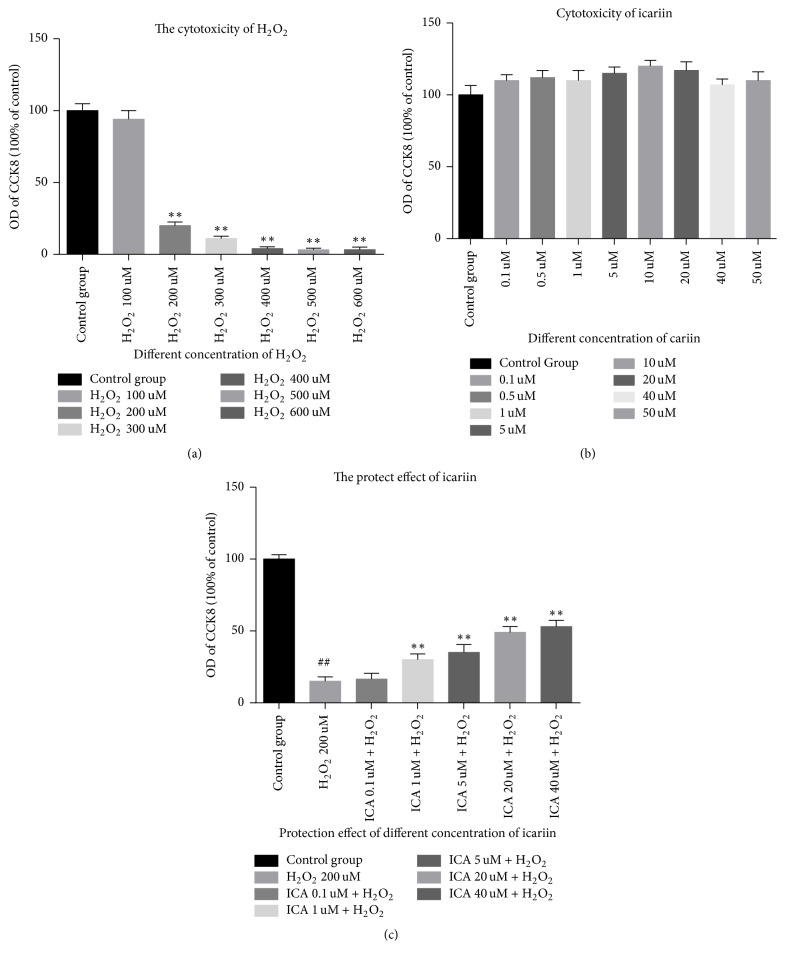
Icariin have no cytotoxicity in NP cells and had a significant protective effect on NP cells exposed to H_2_O_2_ at the concentration of 200 uM and this protective effect was attenuated by the PI3K/AKT pathway inhibitor LY294002. (a) H_2_O_2_ led to obvious cell death at the concentration of 200 uM to 600 uM. Results are presented as mean ± SD (^*∗∗*^*p* < 0.01 versus control group). (b) Icariin alone has no effect on the viability and proliferation of NP cells at concentrations up to 50 *μ*M. (c) Icariin protects NP cells against H_2_O_2_-induced toxicity (^##^*p* < 0.01 versus control group, ^*∗∗*^*p* < 0.01 versus H_2_O_2_ group).

H_2_O_2_ can lead to cell dead and icariin alone has no cytotoxicity in NP cells; what is more, icariin can protect against the H_2_O_2_-induced cytotoxicity. Our date revealed the best concentration of H_2_O_2_ which can lead to a favourable cell apoptosis is 200 *μ*M (Figure 1(a)). The viability and proliferation of cells treated with icariin for 24 h at various concentrations (0.1, 0.5, 1, 5, 10, 20, 40, and 50 *μ*M) were not significantly different (*p* > 0.05) compared to the control group (Figure 1(b)). In addition, icariin provided significant protection when NP cells were exposed to 200 *μ*M H_2_O_2_ (Figure 1(c)). The results of the above are detected by CCK8.

### 3.2. The Apoptosis Rate of Different Tests

**Figure 2:**
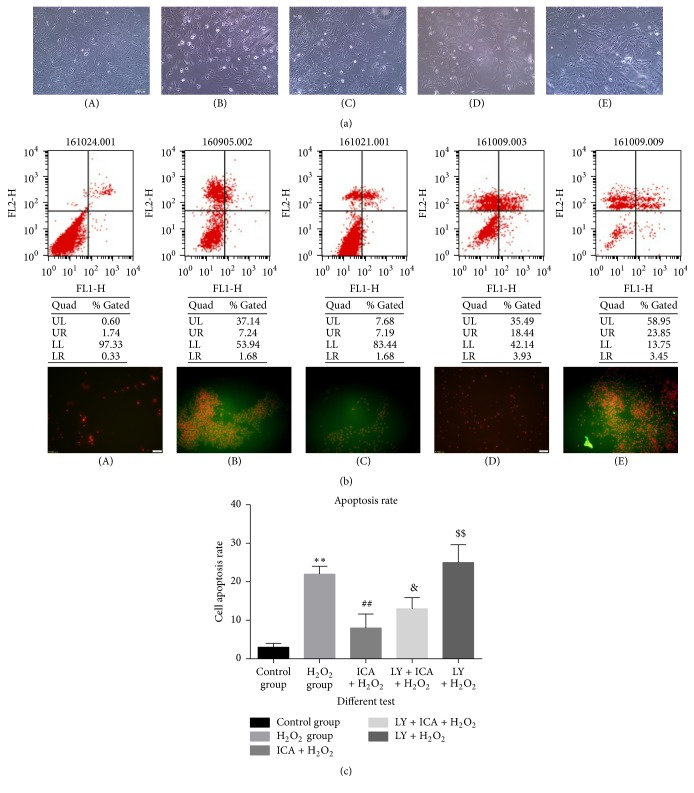
The sectionalization: (A) blank control. (B) 200 *μ*M H_2_O_2_. (C) 200 *μ*M H_2_O_2_ + 20 *μ*M icariin (Ica). (D) 200 *μ*M H_2_O_2_ + 20 *μ*M Ica. (E) 200 *μ*M H_2_O_2_ + 20 *μ*M Ica + 25 *μ*M LY294002. (a) Phase-contrast light microscopy observations: the cell number of group (B) is decreased, cells are more shrunk, and there were many round dead cells. In group (C), there were fewer dead cells and the cell morphology was more normal than in group (B). However, in group (D), cells shrank and there were many dead cells. (b) Apoptosis detected by flow cytometry and fluorescence microscope: the extent of apoptosis significantly declined in group (C) compared with group (B). In group (D), the extent of apoptosis increased compared with group (C). Group (E) exhibited the most apoptotic cells of all groups. (c) Apoptosis rate: the apoptosis rate of H_2_O_2_ group increased significantly and the cytotoxicity of H_2_O_2_ is weakened by icariin (^*∗∗*^*p* < 0.01 versus control group; ^##^*p* < 0.01 versus H_2_O_2_ group). In addition, the lock of PI3/AKT pathway made a dent in the protection effect of icariin (^&^*p* < 0.05 versus group (C)). Group (E) (25 *μ*M LY294002 + 200 *μ*M H_2_O_2_): this group exhibited more apoptotic cells (^$$^*p* < 0.01 versus group (D)).

The protective effect was weakened by a 1 h pretreatment with 20 *μ*M of the PI3K/Akt pathway inhibitor, LY294002 (Figure 2). As shown in Figure 2, cells treated with H_2_O_2_ had a smaller size than control cells and a high percentage of dead cells in the population. This observation was confirmed by fluorescence microscopy and flow cytometry after Annexin V/PI staining of the cells (Figure 2(a)). Morphologic changes and apoptosis were also evident by fluorescence microscopy and flow cytometry with Annexin V/propidium iodide staining (Figure 2(b)). When cells were pretreated with icariin, the number of dead cells was decreased and cell morphology was similar to that of control cells. Groups (D) (200 *μ*M H_2_O_2_ + 20 *μ*M Ica) and (E) (200 *μ*M H_2_O_2_ + 20 *μ*M Ica + 25 *μ*M LY294002) reveal that the cells became shrunk and there were many dead cells. This revealed that the protection by icariin was prevented by inhibiting the PI3K/Akt pathway (*p* < 0.05).

### 3.3. The Intracellular ROS Rate of Different Tests

**Figure 3:**
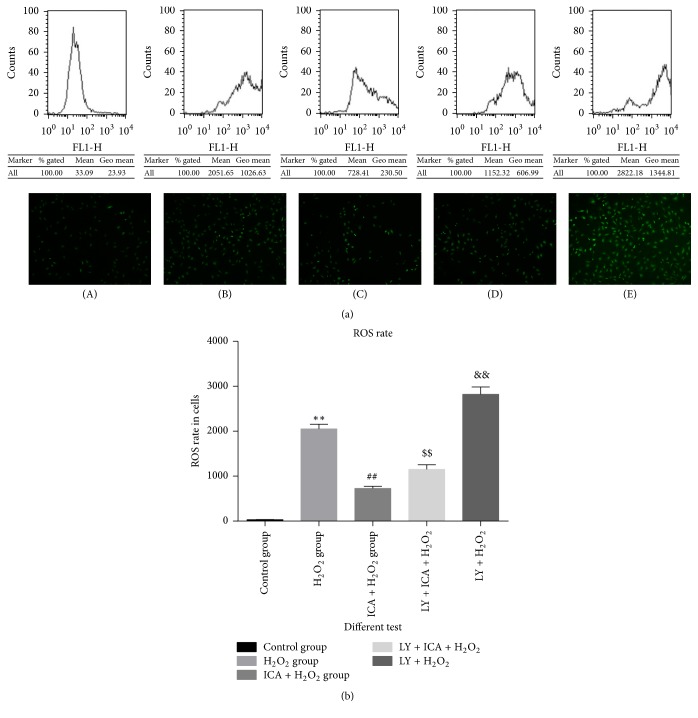
The sectionalization: (A) blank control. (B) 200 *μ*M H_2_O_2_. (C) 200 *μ*M H_2_O_2_ + 20 *μ*M icariin (Ica). (D) 200 *μ*M H_2_O_2_ + 20 *μ*M Ica. (E) 200 *μ*M H_2_O_2_ + 20 *μ*M Ica + 25 *μ*M LY294002. (a) Flow cytometry detection and fluorescence microscope observations: the intracellular ROS dye of group (B) rises. In group (C), there were fewer intracellular ROS than in group (B). However, in groups (D) and (E), intracellular ROS dye increased. (b) ROS rate: the ROS rate of H_2_O_2_ group increased significantly and the cytotoxicity of H_2_O_2_ is weakened by icariin (^*∗∗*^*p* < 0.01 versus control group; ^##^*p* < 0.01 versus H_2_O_2_ group). In addition, the lock of PI3/AKT pathway made a dent in the protection effect of icariin (^$$^*p* < 0.01 versus group (C)). Group (E) showed the highest ROS levels among all groups, which indicates LY294002 acts synergistically to H_2_O_2_ in elevating intracellular ROS (^&&^*p* < 0.01 versus group (B)).

With the different interventions of the (A)–(E) groups, ROS levels in NP cells varied in parallel with the extent of apoptosis (Figure 3). Similar results were observed by both fluorescence microscopy and flow cytometry. Compared to the control group, cells treated with 200 *μ*M H_2_O_2_ showed a significant increase of intracellular ROS levels (*p* < 0.05). Cells pretreated with icariin showed intracellular ROS levels significantly lower compared to group (B) (*p* < 0.05). Intracellular ROS levels of group (D) cells (200 *μ*M H_2_O_2_ + 20 *μ*M Ica +25 *μ*M LY294002) were risen compared to group (C) (*p* < 0.05), which demonstrates that the PI3K/AKT pathway participates in the process. Group (E) cells (25 *μ*M LY294002 + 200 *μ*M H_2_O_2_) showed the highest ROS levels among all groups (*p* < 0.05), which indicates that blocking the PI3K/Akt pathway itself can be deleterious for the cells.

### 3.4. The Activation of PI3K/AKT Pathway

**Figure 4:**
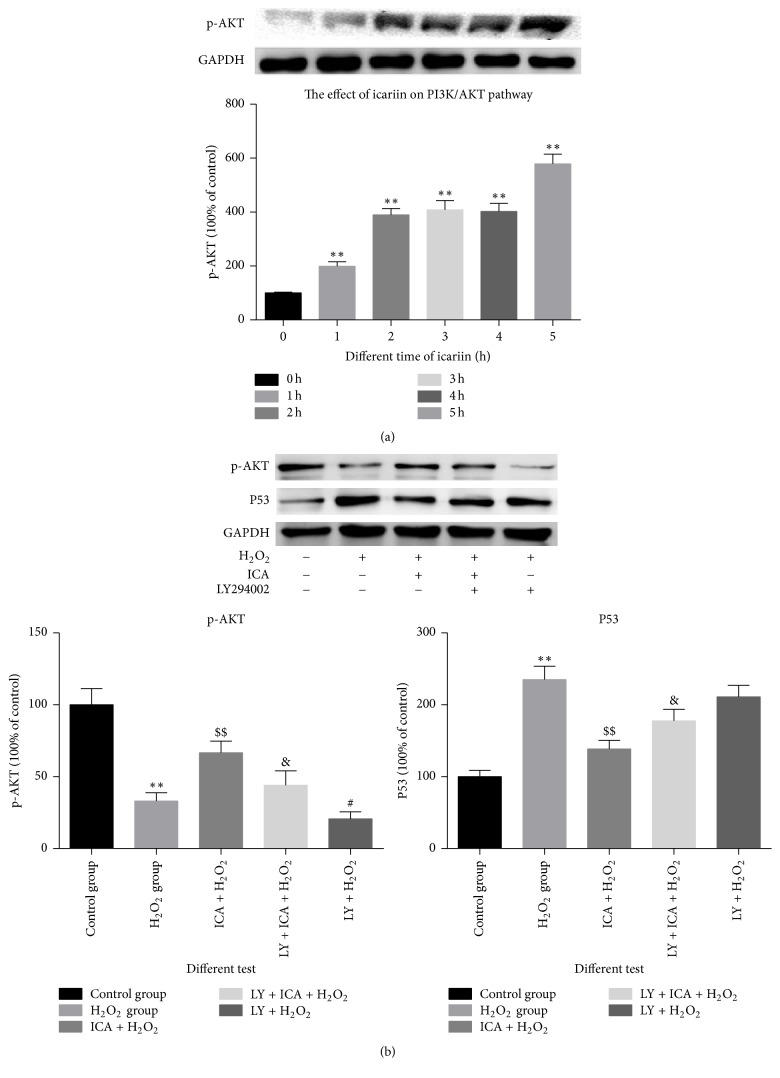
The effect of icariin on the PI3K/Akt pathway. (a) Prolonged culturing of NP cells with icariin increases the expression of p-Akt (^*∗∗*^*p* < 0.01 versus control group). (b) p-Akt and p53 levels in NP cells treated with different agents (^*∗∗*^*p* < 0.01 versus control group; ^$$^*p* < 0.01 versus H_2_O_2_ group; ^&^*p* < 0.05 versus group (C); ^#^*p* < 0.05 versus group (B)). Data are expressed as means ± SD (*n* = 3).

We observed a time-dependent activation of the PI3K/AKT pathway when NP cells were treated with icariin (*p* < 0.05) (Figure 4). Also, with different interventions, p-AKT and p53, the iconic molecules of PI3K/AKT, showed the inverse variation trendy with apoptosis rate, indicating that the activation of PI3K/AKT is a protection factor in the H_2_O_2_-induced apoptosis (Figure 4(b)).

### 3.5. The Expression of Proteins in Apoptosis Pathway

**Figure 5:**
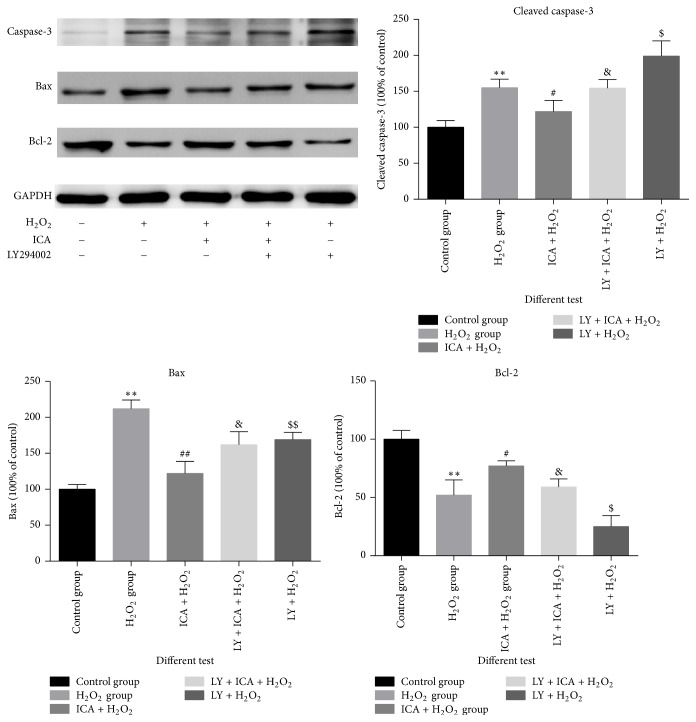
Icariin protects nucleus pulposus (NP) cells from H_2_O_2_-induced apoptosis. Bcl-2, Bax, and caspase-3 were detected by Western blotting. Bcl-2 levels are descended in NP cells treated with H_2_O_2_ (^*∗∗*^*p* < 0.01 versus control group) and Bcl-2 levels are increased in NP cells treated with H_2_O_2_ and icariin when compared with the H_2_O_2_ alone group (^#^*p* < 0.05 versus H_2_O_2_ group) and decreased when the PI3K/Akt pathway is blocked with LY294002 (^$^*p* < 0.05 versus group (C)). Caspase-3 and Bax are decreased in icariin-treated cells (^*∗∗*^*p* < 0.05 versus control group). The protective effect of icariin could be impaired by the presence of LY294002 to block the PI3K/Akt pathway (^##^*p* < 0.01 versus H_2_O_2_ group, ^&^*p* < 0.05 versus group (C)). In addition, LY294002 alone exacerbates H_2_O_2_-induced damage (^$$^*p* < 0.01, ^$^*p* < 0.05 versus H_2_O_2_ group).

The expression of caspase-3, Bax, and Bcl-2 proteins is shown in Figure 5. The antiapoptosis protein bcl-2 decreased when tested with H_2_O_2_ and increased when pretreated with icariin and decreased when the PI3K/AKT pathway was blocked (*p* < 0.05). In contrast, proapoptotic proteins caspase-3 and Bax were decreased in icariin-treated cells. Together, these results showed that icariin had a significant protective effect when NP cells were exposed to H_2_O_2_ and this protection could be impaired by LY294002.

## 4. Discussion

Intervertebral disc degeneration is the most important reason for LBP. There are extensive reports about the pathogenesis of spinal degeneration and the primary therapies currently in use. These include Western medicine, surgical operations, and IVD tissue engineering [11–13]. However, there are few reports on the effect of traditional Chinese medicines, such as icariin, on NP and annulus fibrosus cells, although, as an old Chinese traditional medicine, icariin has been reported to benefit osteogenesis in vivo [14], accelerate the differentiation of osteoblast and mesenchymal stem cells [15], and protect neurocytes [16]. Additional effects are described in numerous review articles [9, 15, 17–24].

The current study is the first report of the effect and the possible mechanism of icariin in NP cells exposed to H_2_O_2_. We believe the mechanism of the protective effect of icariin may involve activation of the PI3K/Akt pathway. Icariin attenuates cigarette smoke-mediated oxidative stress in human lung epithelial cells [17], inhibits neurotoxicity in PC12 cells [16], and activates rat bone marrow cells [15]. These results suggest an activation effect of icariin on the PI3K/Akt pathway.

The IVD has no blood vessels to provide nutrition. Thus, degeneration will ensue if it is exposed to acid or oxidative stress. There are reports that oxidative stress caused by H_2_O_2_ can lead to apoptosis of NP cells [25–33]. In this work, we chose H_2_O_2_ to simulate the physiopathological environment with oxidative stress in vitro, because, actually, the NP cells have to face the oxidative stress in vivo. We chose the concentration of 200 *μ*M because this is the lowest concentration that can induce apoptosis of NP cells. We found that, even at 600 *μ*M of H_2_O_2_, NP cells presented the state of necrosis and that, as the concentration of H_2_O_2_ increased from 200 *μ*M to 600 *μ*M, the NP cells showed similar apoptosis state. Related results had also been measured by the methods of CCK8.

Then we found that icariin provides substantial concentration-dependent protection to NP cells exposed to H_2_O_2_ by means of the CCK8 assay. The greatest protection was seen at 40 *μ*M when these cells were exposed to 200 *μ*M H_2_O_2_. Protection could also be observed by light microscopy. Importantly, icariin alone elicited no cytotoxic effect on NP cells at concentrations up to 50 *μ*M.

We used Western blotting to explore the pathways activated in NP cells treated with icariin. We observed an increase in p-Akt, which is the active form in the PI3K/Akt signaling pathway. Because icariin activated the PI3K/AKT pathway in NP cells, we conducted a series of experiments including measurements of ROS levels, apoptosis, apoptosis-related molecules (caspase-3, Bcl-2, and Bax), and the proteins included in PI3K/AKT pathway such as p-AKT and p53. Changes in each of these parameters reflected the protective effect of icariin when NP cells were exposed to 200 *μ*M H_2_O_2_. Importantly, all changes were diminished in cells treated with LY294002, an inhibitor of PI3K. Although the results with a single pharmacologic agent are insufficient to conclude that the PI3K/Akt pathway is the most important or only mechanism involved in the protective effect of icariin, it is clear that this pathway is a factor. The potential role of other pathways and cytokines requires more research, such as MAPK [34, 35] and HIF-1*α* [36] and NF-kappaB and AF-1 [37],which reportedly can be stimulated by icariin. Furthermore, the extracellular matrix of NP cells has not been examined and there may be some differences between the control and experimental groups.

In summary, this is the first report demonstrating the protective effect of icariin on NP cells exposed to H_2_O_2_. In addition, we have proposed a possible mechanism for this protection involving the PI3K/Akt pathway. These results may suggest new approaches to prevent spinal degeneration and decrease the damage that is caused by the imbalance of oxidative stress.
